# The Chilean exposome-based system for ecosystems (CHiESS): a framework for national data integration and analytics platform

**DOI:** 10.3389/fpubh.2024.1407514

**Published:** 2024-07-24

**Authors:** Patricia Matus, Alejandro Sepúlveda-Peñaloza, Keneth Page, Claudia Rodríguez, Marcela Cárcamo, Francisco Bustamante, Marcela Garrido, Cinthya Urquidi

**Affiliations:** Department of Epidemiology and Health Studies, Medicine Faculty, Universidad de los Andes, Santiago, Chile

**Keywords:** exposome, data integration, ecosystems, systems of system, public health

## Abstract

The double burden of diseases and scarce resources in developing countries highlight the need to change the conceptualization of health problems and translational research. Contrary to the traditional paradigm focused on genetics, the exposome paradigm proposed in 2005 that complements the genome is an innovative theory. It involves a holistic approach to understanding the complexity of the interactions between the human being’s environment throughout their life and health. This paper outlines a scalable framework for exposome research, integrating diverse data sources for comprehensive public health surveillance and policy support. The Chilean exposome-based system for ecosystems (CHiESS) project proposes a conceptual model based on the ecological and One Health approaches, and the development of a technological dynamic platform for exposome research, which leverages available administrative data routinely collected by national agencies, in clinical records, and by biobanks. CHiESS considers a multilevel exposure for exposome operationalization, including the ecosystem, community, population, and individual levels. CHiESS will include four consecutive stages for development into an informatic platform: (1) environmental data integration and harmonization system, (2) clinical and omics data integration, (3) advanced analytical algorithm development, and (4) visualization interface development and targeted population-based cohort recruitment. The CHiESS platform aims to integrate and harmonize available secondary administrative data and provide a complete geospatial mapping of the external exposome. Additionally, it aims to analyze complex interactions between environmental stressors of the ecosystem and molecular processes of the human being and their effect on human health. Moreover, by identifying exposome-based hotspots, CHiESS allows the targeted and efficient recruitment of population-based cohorts for translational research and impact evaluation. Utilizing advanced technologies such as Artificial Intelligence (AI), Internet of Things (IoT), and blockchain, this framework enhances data security, real-time monitoring, and predictive analytics. The CHiESS model is adaptable for international use, promoting global health collaboration and supporting sustainable development goals.

## 1 Introduction

The persistently high burden of non-communicable diseases (NCDs) worldwide (1, 2) highlights the shortcomings of preventive interventions in real-world settings. After birth, individuals live in a complex ecosystem where environmental factors influence their risk of developing diseases and the effectiveness of interventions evaluated in controlled experiments (3, 4). While a small proportion of diseases are genetic in origin, the majority are attributable to environmental factors (5–7). Similarly, predictive models in precision medicine exhibit significant unexplained variability, which is attributed to contextual factors such as social determinants and lifestyles (8, 9).

Studies have often focused on individual factors or specific environmental exposures. In 2005, Wild proposed the novel exposome paradigm to complement the human genome in cancer research, which consider the totality of exposures throughout a person’s life and their complex interactions, including chemical, biological, psychosocial, and lifestyle factors (10). Subsequently, Wild stated that the exposome approach comprises the external (general and specific) and the internal exposome. The external exposome includes all external environmental or contextual factors affecting an individual, such as pollutants, lifestyle, and socioeconomic status. The internal exposome encompasses biological responses and processes (the omics) within the body, like metabolism, immune response, and the microbiome (11). Thus, exposome studies offer a more detailed and comprehensive understanding of the complex interactions between environmental exposures and their influence on internal biological processes and vice versa, contributing to the development of more effective strategies for disease prevention and public health interventions.

Complementing the exposome theory with the One Health approach offers a powerful novel paradigm for advancing public health. The One Health approach recognizes that human health is closely linked to the health of animals and their shared environment. By emphasizing the interconnectedness of human, animal, and environmental health, both the One Health approach and the exposome concept benefit from interdisciplinary collaboration and integrated data systems. This synergy enables comprehensive research and public health interventions, enhances disease surveillance, informs public health policies, and supports the development of innovative strategies to tackle complex health challenges across species and ecosystems. Understanding disturbances in one species can provide critical insights into promoting health in others, leading to a more holistic and effective approach to public health (12, 13).

Several exposome-based initiatives have emerged, including the HELIX, HERCULES, EXPOsOMIC, and the European Human Exposome Network (EHEN) (14–19). Most of these projects focus on European populations with diverse geographical scopes, targeting specific diseases or health outcomes, such as early-life development, cardiovascular and metabolic diseases, chronic respiratory diseases, or major chronic diseases. These initiatives typically involve interdisciplinary collaboration to develop tools for exposome research by integrating data from previously recruited birth cohorts. Additionally, these projects are addressing challenges such as data privacy, diverse data types, temporal variation, and geographic data scales through the development of technological solutions and robust protocols.

Although mega-cohort studies, including biobanks, provide the framework for exposomic research, they require substantial investment. Latin American (LATAM) and other developing countries have different socioeconomic contexts and scarce research resources. Additionally, assembling population cohorts is expensive. Conversely, administrative data that is routinely collected provide insights into societal trends, resource allocation, and program effectiveness, and they are valuable for policy and decision-making across various sectors. Moreover, the Pan American Health Organization coordinated public health efforts to strengthen epidemiological surveillance in LATAM countries, providing a platform for member countries to share epidemiological data and information.

Most countries face a double burden of diseases, which refers to the coexistence of communicable (infectious) and NCD within a population, community, or individual. This phenomenon is particularly relevant in transitional or developing societies that are undergoing rapid social, economic, and demographic changes (20). Chile, a LATAM country, has demonstrated a sustained rapid economic growth over the last few decades. However, a socioeconomic gap persists, with income inequality being a notable concern. The epidemiological transition in Chile reflects a shift from a predominance of infectious diseases to an increasing burden of NCD, which is often associated with lifestyle changes. The coexistence of communicable diseases and NCDs highlights the complex health landscape during Chile’s economic evolution (21).

The purpose of this communication is to describe the conceptual model of the Chilean exposome-based system for ecosystems CHiESS project, which is complemented by the ecological and One Health approaches, and the technological development of a platform that allows the integration of existing administrative data, operationalization of exposome, and evaluation of the exposome impact on health outcomes.

## 2 CHiESS description

### 2.1 Aim of CHiESS

The primary purpose of the CHiESS project is to develop an intelligent technological platform that allows exposome operationalization and to investigate its effects on human health using administrative data. This platform will also serve as a foundation for integration with primary omics data. Thus, we aim to better understand the etiology of the disease and the translation of scientific evidence of both individual and population interventions to real-world settings.

The specific objectives of the CHiESS project are as follows:

1. To build a dynamic and automated system that allows the integration, analysis, and visualization of a wide range of environmental stressors and individual data.
2. To operationalize and map the external and internal exposomes for identifying and monitoring novel hotspots.
3. To study the exposome contribution to the disease’s etiology and the impact of preventive interventions.

The protocol was approved by the Scientific Ethics Committee of the Universidad de los Andes, Chile.

### 2.2 Administrative data sources

Leveraging available administrative data is a differentiating focus of CHiESS. Administrative data sources encompass a wide range of information that governmental and organizational entities collect for administrative purposes. These sources include the following (Table 1).

**Table 1:** Description of potential data sources for CHiESS.

Description	Data sources	Datasets	Level of measurement	Temporal scale
Government agencies: National, regional, and local government agencies collect data for various functions, such as census and national surveys.	Instituto Nacional de Estadística	Population projections	National Regional Communal	Annually
	Banco Central de Chile	Economic indicators	National	Daily
	Sistema de Información Nacional Municipal	Economic indicators	Communal	Annually
	Departamento de Estadísticas e Información de Salud - Ministerio de Salud	Hospital discharges Emergency care Deaths Births	Communal	Monthly Annually
	Ministerio de Salud	National Health Survey Quality of Life Survey	Individual	Every nine years
	Ministerio de Desarrollo Social y Familia	National Socioeconomic Characterization surveys	Individual	Every three years
Health care system: Public and private hospitals and health insurance providers maintain data on patient demographics, medical treatments, and outcomes.	Electronic medical record registration system	Electronic medical records for health control and care	Individual	Monthly
	Superintendencia de Salud	Beneficiary population of public and private systems	National Regional Communal	Annually
Epidemiological surveillance systems: Health-related data is systematically collected to monitor the spread of diseases, identify trends, detect outbreaks early, and inform the public regarding health interventions and decisions.	Epivigila- Ministerio de Salud Portal de datos abiertos – Ministerio de Ciencias	Circulation of respiratory viruses Vaccination Notifiable diseases	Individual	Daily Monthly Annually
Educational institutions: Schools, colleges, and universities gather data on student enrollment, academic performance, and other educational metrics.	Datos abiertos – Centro de estudios Ministerio de Educación	Students and preschoolers Educational establishment cadastre	Educational establishments	Annually
Social services: Agencies responsible for social welfare, housing, and employment collect data on individuals and families receiving assistance.	Ministerio de Desarrollo Social y Familia	Social development report	National Regional	Annually
Employment and labor departments: These agencies collect data on employment, wages, and workforce demographics.	Instituto Nacional de Estadística	The National Employment Survey	National Regional	Annually
Environmental agencies: Organizations responsible for ecological regulation collect data on pollution levels (air, water, and soil), emissions, and environmental compliance	Sistema de Información Nacional de Calidad de Aire del Ministerio del Medio Ambiente.	Air pollution	National Regional Communal	Daily
	Dirección Meteorológica de Chile	Meteorological parameters such as temperature, wind, and precipitation	National Regional Communal	Daily
	Superintendencia de Servicios Sanitarios	Drinking water quality	National Regional Communal	Monthly
	Geoquímica de Relaves, Servicio Nacional de Geología y Minas.	Tailings	National Regional Communal	Annually
Transportation authorities: Agencies managing transportation infrastructure gather data on traffic patterns, public transit usage, and road conditions.	Observatorio de Seguridad vial – Comisión Nacional de Seguridad en el Tránsito	Traffic accidents	National Regional	Annually

### 2.3 General research questions and conceptual model of CHiESS

The CHiESS project raises the following three general research questions, which address priority current diseases according to biological plausibility and the current state of knowledge:

- What environmental exposure patterns, temporal accumulation of patterns, interaction of patterns, or mechanisms contribute to the diseases?
- Can omics signals or biomarkers of the health effects of multiple and prolonged exposures to environmental stressors be identified?
- What individual- or population-based interventions could be targeted using the exposome paradigm?

Consequently, the integration and analysis of CHiESS data are founded on specific hypotheses that will be derived from the previously formulated research questions, focusing on certain diseases such as diabetes, cancer, and obesity, among others. Furthermore, data integration and analytics within CHiESS adhere to an agnostic approach, which is a hypothesis-free method aimed at uncovering new disease etiologies through omics and non-omics technologies, devoid of specific preconceptions or predetermined conclusions.

**Figure 1:**
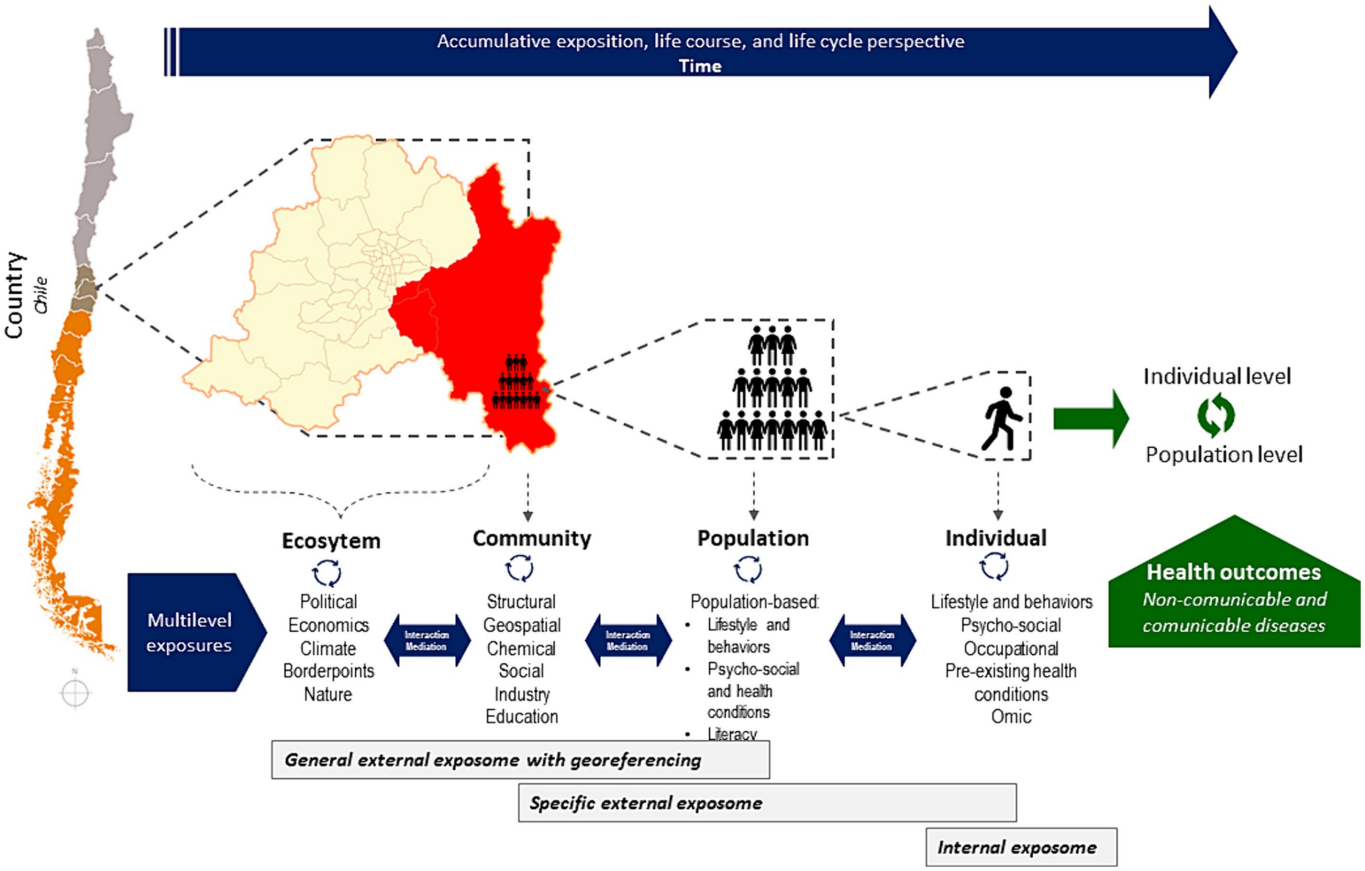
Conceptual model.

Based on the ecological theory, which examines patterns and processes in nature at different levels of organization (from individual units to larger systems), and considering the geospatial and administrative division of Chile, the CHiESS conceptual model consists of four levels of exposure: (1) ecosystem, (2) community, (3) population, and (4) individual (Figure 1). The ecological model is justified as it allows for a comprehensive understanding of environmental and biological interactions at different scales, which is essential for investigating the multifactorial nature of health outcomes. Additionally, the ecological approach permits the conceptualization and operationalization of shared and non-shared exposures, where shared exposures pertain to the general external exposome in specific geographic areas, and non-shared exposures relate to individual lifestyles or specific external exposome. Furthermore, the One Health approach, which recognizes the interconnectedness of human, animal, and environmental health, is integral to CHiESS, providing a holistic framework to address complex health issues.

#### 2.3.1 Ecosystem

Continental Chile is divided into 16 regions, each with distinct climates, soils, economic activities (e.g., mining and agriculture industry), and biodiversity due to the country’s extensive geographical length. For instance, the northern regions are characterized by deserts, arid areas, and high peaks of the Andes mountains, while the southern regions, feature humid jungles to glacial areas. The central regions are predominantly urbanized and engage in intensive agriculture. Thus, the ecosystem encompasses the biotic organisms and abiotic factors of each region, including political, economic, and climate, denoting shared exposures of the general external exposome. This level can be extended to a broader area range, such as two or more countries or WHO regional areas (Figure 1).

#### 2.3.2 Community

Chile is further divided into 346 communes, the smallest unit of local administration, managed by municipalities. At this level, a community includes all populations of different species coexisting in a location, such as mammals, birds, and other species that can spread diseases. The community level in CHiESS, includes geographical characteristics, species distribution, and behavior (including zoonotic vectors and domestic and productive animals), as well as social, educational, or chemical exposures at the commune level, denoting the shared exposures of the general external exposome, but within a smaller areas and including other species (Figure 1).

#### 2.3.3 Population

According to the ecological organization theory, this level examines all individuals of the human species in a specific area at a given time. Moreover, given the variations in population density across communes, different ecosystems emerge. The population level in CHiESS focuses on human-specific exposures and behaviors (in contrast to the communal level, which includes other species and geographic factors), constructing aggregated indicators of exposure to environmental contaminants, lifestyles, psychosocial, economic, and health-related factors, denoting the specific external exposome of unshared exposures but at the population level (Figure 1).

#### 2.3.4 Individual

This level focuses on a single organism and its physiology, behavior, and adaptations. At the individual level, exposures assessed at the population level will be evaluated on a personal scale, denoting unshared individual exposures of the specific external exposome (Figure 1).

At the individual level, CHiESS includes the internal exposome represented by omics data, which will allow for the identification of biomarkers and omics signals associated with prolonged and multiple exposures to environmental stressors. By linking these biomarkers with specific exposure patterns identified at higher levels of the model (ecosystem, community, and population), CHiESS can elucidate the biological pathways affected by environmental stressors. This approach enables the identification of molecular signatures that indicate the health effects of complex exposure scenarios, providing insights into the mechanisms of disease development.

#### 2.3.5 CHiESS outcomes

The project’s purpose is to provide evidence for both local public policies and precision medicine concerning NCD, re-emerging and emerging infectious diseases, and for countries confronting the double burden of disease. The CHiESS addresses outcomes for both NCD and communicable diseases, considering their etiology and transmission. Health effects will also be assessed at various levels, with outcomes defined at both the individual and population levels (green arrows in Figure 1).

The 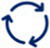 symbol in Figure 1 denotes the complex interaction between the multilevel exposures within each level and between levels. The 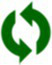 symbol corresponds to the recursiveness of health effects (i.e., how individual outcomes contribute to population indicators and vice versa).

CHiESS acknowledges the cumulative and dynamic nature of exposures by investigating how exposures accumulate and interact across different life stages, from prenatal development through childhood, adolescence, adulthood, and into old age, illustrated by the arrow at the top indicating time in Figure 1. Additionally, the accumulative exposure over time can occur at the four levels of CHiESS. For example, considering the economic evolution of Chile and within its region (ecosystem), changes in biodiversity or social factors within the commune (community), shifts in social dynamics of the population (population), or changes in individual lifestyle choices (individual).

By mixing the ecological and One Health approaches to the exposome, CHiESS aims to capture the complex interplay between environmental, biological, and social factors influencing health. This comprehensive approach is critical for developing targeted interventions and informing public health strategies responsive to the unique contexts of different regions and communities. Thus, the CHiESS conceptual model can be applicable to other countries. Additionally, the ecological approach allows for the conceptualization and further operationalization of shared exposures, which pertain to the general external exposome, and non-shared exposures, which relate to individual lifestyles.

The CHiESS model systematically integrates data across four levels of exposure: ecosystem, community, population, and individual. This multilevel approach allows for the comprehensive analysis of environmental exposure patterns and their temporal accumulation. By examining interactions between various exposures (e.g., socioeconomic, chemical, and biological), CHiESS aims to identify mechanisms that contribute to diseases. The model’s ecological basis facilitates the exploration of how different exposures interact within and across levels to influence health outcomes. For example, by analyzing data at the community level, the model can reveal how local environmental factors, such as pollution and social stressors, combine and accumulate over time to impact health.

Finally, the CHiESS model provides a framework for defining environmental health priorities, guiding both cohort recruitment and the development of targeted interventions. By characterizing exposures at multiple levels (ecosystem, community, population, and individual), CHiESS can identify high-risk areas and populations for targeted interventions. For instance, at the population level, aggregated indicators of exposure can inform public health strategies, while at the individual level, personalized interventions can be designed based on specific omics profiles. The model’s ability to integrate and analyze diverse data types allows for the identification of intervention points that can mitigate the impact of harmful exposures, ultimately contributing to precision public health.

### 2.4 CHiESS technological framework

**Figure 2:**
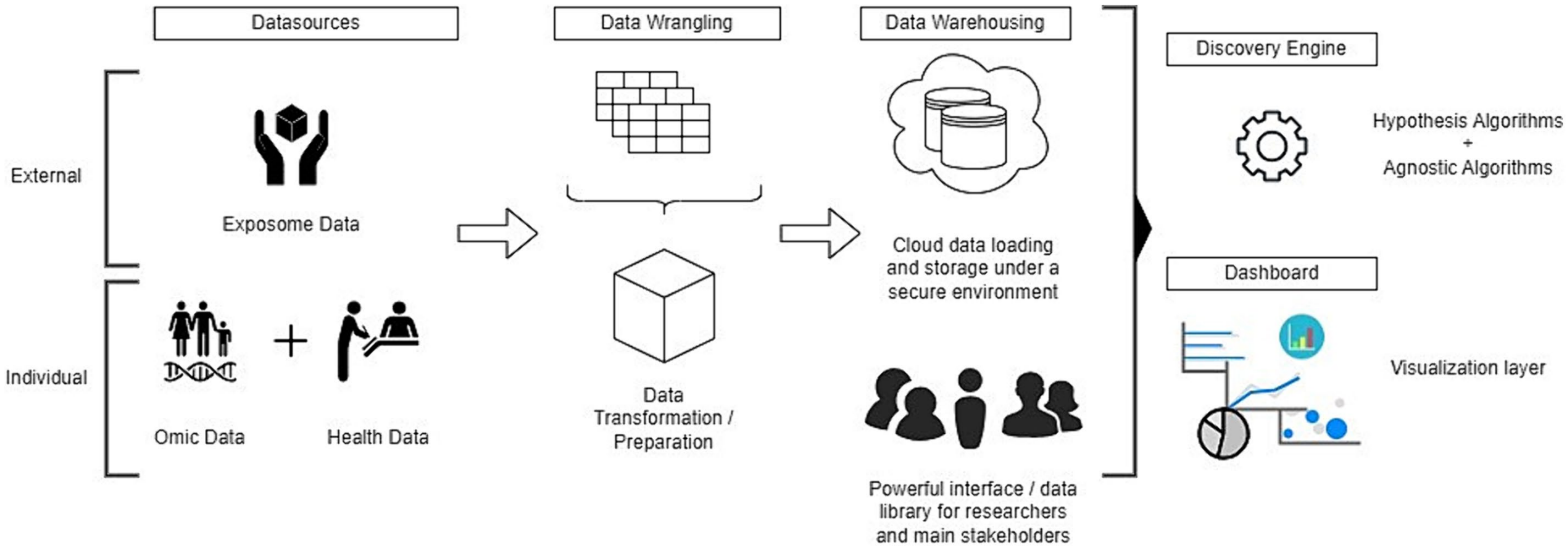
The technological framework of the platform.

The leading architecture and data interactions in CHiESS for the platform are based on five layers that evolve in a dynamic and robust environment: (1) diverse data sources, (2) data wrangling, (3) data warehousing, (4) discover engine, and (5) data dashboard (Figure 2).

The CHiESS platform architecture seamlessly integrates external exposome information with individual omics and healthcare provider data. This first layer manages a diverse array of external data sources from government agencies, epidemiological surveillance systems, educational institutions, social services, employment and labor departments, transportation authorities, environmental agencies, and statistical agencies. The data may include relevant information such as air quality indices, pollution levels, socioeconomic indicators, lifestyle information, and zoonotic vectors. This layer also interfaces seamlessly with individual omics data from healthcare systems, clinical data, electronic health records, lifestyle assessments, and other original studies. Additionally, it incorporates genomics, epigenomics, transcriptomics, metabolomics, and proteomics to provide a complete view of an individual’s biological makeup. The collection of this data will be challenging because the information should be reconciled cohesively.

The second layer addresses the data heterogeneity with a robust data wrangling and preparation process that harmonizes the diverse datasets. This ensures consistency and quality of the integrated datasets, addresses potential discrepancies, and enhances the overall reliability of the exposome studies. The architecture employs sophisticated data integration and transformation techniques, ensuring that disparate data types are harmonized and normalized to facilitate meaningful correlations and analyses across dimensions. This transformation process involves careful consideration of temporal, spatial dimensions, standardization, and normalization, as well as data quality checks to eliminate potential biases.

A secure cloud-based data warehousing layer efficiently stores the pre-processed data to ensure an efficient and protected environment for sensitive information. This layer serves as a dynamic interface that facilitates efficient storage and allows scalable and flexible data management. Researchers, stakeholders, and policymakers benefit from this central repository that streamlines collaborative efforts and ensures data integrity.

In the final dual-layer, an engine for advanced algorithms operates in tandem with a visualization tool to provide a comprehensive platform for analysis and interpretation. This final engine layer extracts patterns and associations from the integrated data via two types of analyses (hypothesis-based and agnostic algorithms). Simultaneously, the visualization tool translates complex findings into interpretable insights, which facilitates comprehension and decision-making. The platform capitalizes on advanced techniques to transform complex data into interpretable insights. A diverse array of visualization methods is employed to present findings in a meaningful, accessible, and effective manner. Graphical representations, such as interactive charts, heatmaps, and network diagrams, offer intuitive insights into patterns and relationships within the integrated datasets. Geographic visualizations overlaying the exposome data on maps facilitate the identification of spatial trends, which is crucial for understanding regional variations in environmental exposures and health outcomes. Furthermore, it incorporates dynamic views that enable historical and real-time exploration of data trends. Thus, stakeholders can focus on specific variables or timeframes which facilitate targeted analyses. This interactive approach enhances user experience and promotes collaboration by allowing diverse stakeholders to contribute their expertise to the exposome studies.

## 3 Project stages development

CHiESS will undergo four consecutive stages in its development into an informatics platform.

### 3.1 Stage one: environmental data integration and harmonization system

The first stage aims to develop the initial data integration and harmonization components. This stage also involves the development of a data integration application and the processing of queries to allow geospatial operationalization of the general external exposome at the ecosystem (regions) and community (commune) levels. Validation at this stage will consist of determining data inconsistency, integrity, and post-transformation accuracy according to data governance policies and privacy regulations.

### 3.2 Stage two: health and omics data integration

The second stage focuses on developing complementary informatics components for integrating and harmonizing data from clinical records and biobanks. This will operationalize the specific external exposome at the population level and the internal exposome at the individual level. Validation at this stage will consist of determining data integrity and post-transformation accuracy in addition to confirming the concordance of systems results with well-known geospatial exposure distribution and omics-biomarkers outcomes.

### 3.3 Stage three: development of advanced analytical algorithms

The third stage involves developing hypothesis-based or supervised and unsupervised agnostic algorithms complemented by causal diagrams. The conceptual model of CHiESS encompasses the operationalization of exposures at different levels of observation and aims to evaluate trends and complex interactions of multiple exposures. Therefore, data-driven analytics will be based on the exposome-wide association study (ExWASs) approach, which considers mixed models for nested or correlated data, weighting for complex survey samples when data comes from national surveys, different types of variable selection such as LASSO or Ridge regressions (22), and mixture analysis such as Weighted Quantile Sum or Bayesian approaches or Gaussian process regression (23). Additionally, causal machine-learning models (24) will complement hypothesis-based association analyses.

The conceptual model of CHiESS also encompasses the operationalization of the external exposome in specific geographic areas and spatiotemporal data linkage. Thus, geospatial techniques to account for spatial autocorrelation and to model the spatial distribution of exposures and health outcomes will be considered, as well as techniques for small-area analysis such as Fay-Herriot and Spatial Fay-Herriot models (25).

### 3.4 Stage four: visualization interface development and population-based cohort recruitment

The fourth stage is focused on developing and implementing visualization and monitoring interfaces for mapping and surveillance hotspots for final users and stakeholders. Moreover, the identification of hotspots at this stage will help determine policy recommendations, plan interventions, and recruit a hotspot-targeted population-based cohort for the measurement of environmental stressors and omics, as well as a final external validation.

## 4 Main challenges

Undoubtedly, CHiESS is an ambitious and large-scale project that presents many challenges, some of which have been identified previously by other initiatives, and others will likely continue to emerge (26–30).

The main challenges are the high dimension integration and mining data with time and spatial dependence structure, dealing with heterogeneity data, and the development of wearable measurement sensors. Fortunately, with the advancements in bioinformatics, big data analytics, artificial intelligence, and machine learning, among others, these challenges can be overcome.

Another challenge for CHiESS is incorporating causal structures in the integrated data, ensuring advanced analytics are appropriately tailored to a robust causal design that tests hypotheses, and understanding the role of confounders. Therefore, epidemiological and clinical reasoning is essential during data integration and analysis. Since CHiESS is initially based on administrative data integration, validating exposure and outcome data measurements is another challenge. The application of known exposure or outcomes biomarkers, complemented by validated questionnaire-based approaches, may overcome this limitation because altered mRNA, proteins, or metabolite levels will reflect specific environmental exposures.

Notwithstanding the aforementioned limitations, matching omics measurements with functionalities (e.g., biomarker development, differentiating exposures from biological responses, investigating mixtures and interactions between agents, and understanding mechanisms for biological plausibility and etiology of diseases) is undoubtedly another challenge for CHiESS due to its cost. Hence, the prospective cohort study design best suits the exposure biomarker approach, providing opportunities for repeat sampling to enable a broader timeframe of exposure assessment and avoid reverse causation by collecting samples before the disease onset. Thus, CHiESS provides criteria for defining environmental health priorities, which will guide priority cohort recruitment as well as research and tailoring omics measurement and target intervention at the population level. A geocoding process to a geospatial dimension is necessary to integrate existing or new data. One of the advantages in Chile is the existence of a unique identifier number for individuals, which facilitates the integration of individual data while adhering to confidentiality standards. On the other hand, due to the multilevel and geospatial conceptual mode of CHiESS, we plan to calculate the geospatial diameter of a commune and match it with individual addresses and thus their exact coordinates, but we will need to define an analysis radius to cross-reference information within a commune. This could lead to issues, for example, where a person lives on the border of a commune and exhibits symptoms of pollution from a neighboring commune that falls within the defined perimeter.

Because CHiESS is based on the One Health ecosystem approach, another challenge is understanding the particularities of each health problem and establishing connections with general processes such as globalization, territorial expansion, migration, job insecurity, vulnerability of populations, environmental degradation, and urbanization (31). Thus, a multidisciplinary team composed of epidemiologists, sociologists, clinicians, environmental health experts, agricultural scientists, ecologists, veterinarians, microbiologists, biostatisticians, and geospatial analysts is essential for the formulation of specific yet holistic hypotheses and data analysis. On the other hand, the technological development will need to be addressed by data scientists to develop algorithms and models for integrating and analyzing diverse datasets, software engineers to develop and maintain the platform’s software infrastructure, computer scientists or informatics specialists to manage the hardware and network infrastructure required for data storage and processing, and a project Manager to coordinate the efforts of the multidisciplinary team and ensure timely progress. Fortunately, innovation and technological development from academia, along with interdisciplinary collaboration and partnerships with industry, make this feasible.

The major challenge of a technology platform integrating health data is ensuring the protection of sensitive information. This includes safeguarding data from authorized access, preventing breaches or leaks, and complying with privacy regulations such as HIPAA (Health Insurance Portability and Accountability Act) in the United States or GDPR (General Data Protection Regulation) in the European Union. In Chile, Law 19.628, also known as the Chilean Data Protection Law (Ley de Protección de Datos Personales), regulates the protection of personal data. It establishes principles and rules for the processing of personal data, including the rights of individuals regarding their data and the obligations of data controllers and processors. Compliance with Law 19.628 is crucial for any technology platform handling health data in Chile, as it sets requirements for data security, consent, notification of data breaches, and other aspects of data protection. Hence, CHiESS contemplates compliance with national and international laws, and it will need to prioritize to ensure the integrity and reliability of the data amidst evolving cybersecurity as part of its implementation.

Finally, other ethical challenges in data integration could arise from the potential mishandling of data, privacy concerns, security risks, data governance, transparency and accountability, legal and regulatory compliance, and the impact on individuals and society. Thus, applying technical measures, legal frameworks, and ethical guidelines will ensure that data integration is conducted responsibly and transparently.

## 5 Discussion

The complexity of human exposome needs to be investigated progressively. To our knowledge, CHiESS is an innovative exposome-based project in LATAM that contextualizes the human exposome and introduces the exposome paradigm in translational research, which can be scaled and contribute to the exposomic research network worldwide. Unlike other initiatives, the CHiESS model is based on the One Health ecosystem approach to include the patterns of other species that contribute to the spread of communicable diseases. Furthermore, CHiESS focuses on the geospatial operationalization of the general external exposome based on administrative and routinely collected data.

The CHiESS project aims to address complex interactions that have been understudied in longstanding health issues, as well as to explore new causal pathways in disease etiology. For example, these may include the complex interactions between socioeconomic status and lifestyle factors in relation to obesity, or the evaluation of the effect of environmental contaminants or toxins, potentially acting through hypothesized endocrine disruptor mechanisms, on the early origins of obesity. Our team has also been working on topics such as suicide in adolescents, anticipating peaks in respiratory illnesses based on environmental factors, and vaccine effectiveness for a better understanding of the impact of interventions in real-world settings, thereby translating evidence to policymakers. Thus, a differentiating factor of CHiESS from other initiatives is the expectation that it can contribute locally to epidemiological surveillance and hotspot identification for policymaker targeting, and as a scalable technological platform for other countries.

The novel CHiESS technological platform involves developing automated and semi-automated systems for data integration, advanced analytics, visualization, and monitoring, which is similar to other platforms (32, 33). This is intended to facilitate meaningful exposome research and empower researchers and stakeholders to derive significant conclusions from integrated data and advanced analytics. We will use Python and R Studio for data analysis and modeling. For the architecture, we will use EC2 to deploy applications on WildFly, MySQL RDS for data, and another development layer on Amazon. Nevertheless, it is essential to carry out a proof-of-concept study to validate the feasibility and effectiveness of data integration and harmonization before it can be fully developed.

In conclusion, the CHiESS is a novel project that will provide an informatics and intelligent platform that will integrate administrative and original target omics data for exposome studies in Latin America, since most Latin America and developing countries are facing the double burden of diseases, contributing to the exposome research network worldwide. Thus, the CHiESS conceptual model is based on the one-health ecosystem approach. It includes environmental stressors and other species’ patterns that contribute to the transmission of infectious diseases and the etiology of NCD. CHiESS will serve as a basis for targeted, evidence-based interventions, efficient and tailored population-based cohort recruitment, and omics measurements.

## Data availability statement

The original contributions presented in the study are included in the article/supplementary material, further inquiries can be directed to the corresponding author.

## Author contributions

PM: Writing – review & editing, Methodology, Conceptualization. AS-P: Writing – review & editing, Software, Methodology, Data curation, Conceptualization. KP: Writing – review & editing, Software, Methodology, Data curation, Conceptualization. CR: Writing – review & editing, Investigation. MC: Writing – review & editing. FB: Writing – review & editing, Supervision. MG: Writing – review & editing, Supervision, Resources. CU: Writing – review & editing, Writing – original draft, Validation, Supervision, Methodology, Funding acquisition, Conceptualization.
